# Prevalence and Antibiotic Resistance Characteristics of Extraintestinal Pathogenic *Escherichia coli* among Healthy Chickens from Farms and Live Poultry Markets in China

**DOI:** 10.3390/ani11041112

**Published:** 2021-04-13

**Authors:** Ming Zou, Ping-Ping Ma, Wen-Shuang Liu, Xiao Liang, Xu-Yong Li, You-Zhi Li, Bao-Tao Liu

**Affiliations:** 1 College of Veterinary Medicine, Qingdao Agricultural University, Qingdao 266109, China; zoudnet@163.com (M.Z.); maping20210219@163.com (P.-P.M.); liuwenshuang0719@163.com (W.-S.L.); liangxiao4000@163.com (X.L.); 2 College of Agronomy, Liaocheng University, Liaocheng 252000, China; lixuyong@lcu.edu.cn; 3 Shandong Veterinary Drug Quality Inspection Institute, Jinan 250022, China; liyouzhi2009@126.com

**Keywords:** characteristics, extraintestinal pathogenic *E. coli*, healthy chickens, multidrug-resistant

## Abstract

**Simple Summary:**

Chicken meat has been proved to be a suspected source of extraintestinal pathogenic *Escherichia coli* (ExPEC), causing several diseases in humans, and bacteria in healthy chickens can contaminate chicken carcasses at the slaughter; however, reports about the prevalence and molecular characteristics of ExPEC in healthy chickens are still rare. In this study, among 926 *E. coli* isolates from healthy chickens in China, 22 (2.4%) were qualified as ExPEC and these ExPEC isolates were clonally unrelated. A total of six serogroups were identified in this study, with O78 being the most predominant type, and all the six serogroups had been frequently reported in human ExPEC isolates in many countries. All the 22 ExPEC isolates were multidrug-resistant and most isolates carried both *bla*_CTX-M_ and *fosA3* resistance genes. Notably, plasmid-borne colistin resistance gene *mcr-1* was identified in six ExPEC isolates, among which two carried additional carbapenemase gene *bla*_NDM_, compromising both the efficacies of the two critically important drugs for humans, carbapenems and colistin. These results highlight that healthy chickens can serve as a potential reservoir for multidrug resistant ExPEC isolates, including *mcr-1*-containing ExPEC.

**Abstract:**

Chicken products and chickens with colibacillosis are often reported to be a suspected source of extraintestinal pathogenic *Escherichia coli* (ExPEC) causing several diseases in humans. Such pathogens in healthy chickens can also contaminate chicken carcasses at the slaughter and then are transmitted to humans via food supply; however, reports about the ExPEC in healthy chickens are still rare. In this study, we determined the prevalence and characteristics of ExPEC isolates in healthy chickens in China. A total of 926 *E. coli* isolates from seven layer farms (371 isolates), one white-feather broiler farm (78 isolates) and 17 live poultry markets (477 isolates from yellow-feather broilers) in 10 cities in China, were isolated and analyzed for antibiotic resistance phenotypes and genotypes. The molecular detection of ExPEC among these healthy chicken *E. coli* isolates was performed by PCRs, and the serogroups and antibiotic resistance characteristics of ExPEC were also analyzed. Pulsed-field gel electrophoresis (PFGE) and Multilocus sequence typing (MLST) were used to analyze the genetic relatedness of these ExPEC isolates. We found that the resistance rate for each of the 15 antimicrobials tested among *E. coli* from white-feather broilers was significantly higher than that from brown-egg layers and that from yellow-feather broilers in live poultry markets (*p* < 0.05). A total of 22 of the 926 *E. coli* isolates (2.4%) from healthy chickens were qualified as ExPEC, and the detection rate (7.7%, 6/78) of ExPEC among white-feather broilers was significantly higher than that (1.6%, 6/371) from brown-egg layers and that (2.1%, 10/477) from yellow-feather broilers (*p* < 0.05). PFGE and MLST analysis indicated that clonal dissemination of these ExPEC isolates was unlikely. Serogroup O78 was the most predominant type among the six serogroups identified in this study, and all the six serogroups had been frequently reported in human ExPEC isolates in many countries. All the 22 ExPEC isolates were multidrug-resistant (MDR) and the resistance rates to ampicillin (100%) and sulfamethoxazole-trimethoprim (100%) were the highest, followed by tetracycline (95.5%) and doxycycline (90.9%). *bla*_CTX-M_ was found in 15 of the 22 ExPEC isolates including 10 harboring additional fosfomycin resistance gene *fosA3*. Notably, plasmid-borne colistin resistance gene *mcr-1* was identified in six ExPEC isolates in this study. Worryingly, two ExPEC isolates were found to carry both *mcr-1* and *bla*_NDM_, compromising both the efficacies of carbapenems and colistin. The presence of ExPEC isolates in healthy chickens, especially those carrying *mcr-1* and/or *bla*_NDM_, is alarming and will pose a threat to the health of consumers. To our knowledge, this is the first report of *mcr-1*-positive ExPEC isolates harboring *bla*_NDM_ from healthy chickens.

## 1. Introduction

*Escherichia coli* is a commensal member of the intestinal tract of warm-blooded animals and most *E. coli* strains are harmless; however, a subgroup has possessed the ability to cause diseases, especially extraintestinal infections caused by the extraintestinal pathogenic *Escherichia coli* (ExPEC) [1]. ExPEC strains could colonize the human gastrointestinal tract, not causing disease; however, diverse infections occur when they enter a normally sterile body site [2]. For example, ExPEC strains have been the leading cause of urinary tract infections primarily affecting women [3] and been also the most common cause of bloodstream infections in humans [4]. Importantly, ExPEC infections would impose a large economic burden due to both medical costs and lost productivity, besides their association with morbidity and mortality [5].

A molecular definition of ExPEC is *E. coli* isolate harboring at least two of five virulence markers: *papA* and/or *papC*, *sfa*/*foc*, *afa*/*dra*, *kpsM* II and *iutA* [6], and this molecular criteria has been widely applied in epidemiological studies. ExPEC strains have been found in various water sources, including environmental water [7], wastewater [8] and drinking water [9]. Retail meats proved to be classic vehicles for several foodborne pathogens, are also commonly contaminated with ExPEC strains [10,11], posing a potential risk to consumers. In recent years, researchers found that human and animal-source ExPEC shared highly similar virulence genes and clonal backgrounds [12,13] and animal-source ExPEC were capable to adhere or invade human intestinal epithelial [14], suggesting that food-producing animals have been a potential source of human ExPEC. Investigations of the ExPEC within poultry were mainly focused on the avian pathogenic *Escherichia coli* (APEC), a subset of ExPEC, from poultry with colibacillosis [15,16] and APEC mainly caused respiratory and systemic disease in poultry; however, the molecular definition criteria of APEC in those studies was different from that of ExPEC [6,17]. Recently, ExPEC isolates within diseased chickens were also reported [18,19]. Notably, the feces of healthy chickens also carried ExPEC isolates [14,20], and the fecal ExPEC isolates could contaminate chicken carcasses at slaughter, including from rupture of the digestive system during processing, and then transmitted to humans by the food chain or direct human-animal contact [21]. However, studies focusing on ExPEC isolates in healthy chickens are still rare, especially in China, which has huge chicken production.

The presence of antibiotic resistance, one of the ten threats to global health for 2019 as determined by the World Health Organization, among ExPEC isolates has been another big concern. Antibiotic resistance genes, such as extended-spectrum β-lactamases (ESBLs)-encoding genes have been reported in ExPEC isolates [22] and ExPEC including those from poultry can also acquire different resistance genes [18], which would inevitably reduce the therapeutic options, increase morbidity and mortality of ExPEC infections, and eventually bring an increased risk to public health [23,24]. Although antibiotics do not select virulent strains such as ExPEC intrinsically [25], the heavy use of antibiotics in food-producing animals could facilitate the dissemination of ExPEC because such pathogens from environment and diseased animals have been often reported to be multidrug resistant (MDR) [7,14]. However, reports focusing on the antibiotic resistance among ExPEC isolates in healthy chickens remain rare, especially in China which has the largest consumption of antibiotics in the world [26].

Therefore, intense research efforts are warranted to fully understand the characteristics of ExPEC isolates from healthy animals to devise new strategies to prevent their dissemination. In this study, we investigated the prevalence of ExPEC isolates among healthy chickens from farms and live poultry markets in 10 cities in China, and the phenotypic and genotypic characteristics of antimicrobial resistance in these ExPEC isolates were also analyzed.

## 2. Materials and Methods

### 2.1. Sampling and Bacterial Isolation

From May 2015 to February 2017, a total of 926 fecal samples were collected from healthy chickens of seven layer farms (371 samples), one white-feather broiler farm (78 samples) and 17 live poultry markets (477 samples) in 10 cities of three provinces (Shandong, Anhui and Shanxi) in China, and 813 samples used in this study were from Shandong province because Shandong has the first largest broiler and layer production in China (Table S1). The white-feather broilers were five-weeks old when the 78 fecal samples were collected, and all the 371 layer fecal samples were from 70-weeks old brown-egg layers before being rejected. All the chickens in the eight farms would be slaughtered within one week after sampling. The 477 fecal samples collected from 17 live poultry markets were from yellow-feather broilers about 12 weeks old sold for consumption and all the yellow-feather broilers from each market were sampled. The chickens in these farms and live poultry markets had been fed with non-medicated feed for at least two weeks before we collected these samples.

A total of 2 g of fecal sample was suspended in 18 ml of trypticase soy broth (BectonDickinson Co., Cockeysville, MD, USA) and incubated aerobically overnight at 37 °C. The broth was then diluted in series of 1:10 and streaked onto MacConkey agar (Qingdao Haibo Microorganism Reagent Co., Ltd., Qingdao, China) followed by incubation for 18 h at 37 °C. Suspected colonies were streaked onto eosin methylene blue (EMB) agar (Qingdao Haibo Microorganism Reagent Co., Ltd., Qingdao, China), and one colony with typical *E. coli* morphology was selected from each sample. The *E. coli* isolates were identified by classical biochemical methods as previously described [27] and confirmed by API 20E system (bioMérieux, Marcy l'Étoile, France). Each farm or live poultry market was sampled only one time when we surveyed the prevalence of antibiotic resistance of *E. coli* and the sampling period covered the four seasons.

### 2.2. Antimicrobial Susceptibility Tests

The minimal inhibitory concentrations (MICs) of 17 antimicrobials—namely, meropenem, cefotaxime, ceftiofur, ampicillin, ciprofloxacin, enrofloxacin, levofloxacin, nalidixic acid, amikacin, gentamicin, kanamycin, streptomycin, tigecycline, doxycycline, tetracycline, florfenicol and fosfomycin—for these isolates were determined by the agar dilution method following the guidelines of the Clinical and Laboratory Standards Institute [28]. The MIC of colistin was determined according to the method of 2017 EUCAST (available at http://www.eucast.org/clinical_breakpoints/ (accessed on 29 June 2018)). The resistant breakpoints for colistin and tigecycline were recommended by the 2017 EUCAST (http://www.eucast.org/clinical_breakpoints/ (accessed on 28 July 2018)), while the breakpoints for the remaining antimicrobials were recommended by the CLSI [28,29]. *E. coli* ATCC 25,922 was used as the control strain. Ceftiofur, florfenicol and tigecycline were from Solarbio Life Sciences Co. (Beijing, China) and the remain antimicrobials used in this study were purchased from China National Institutes for Drug Control (Beijing, China).

### 2.3. Detection of Antibiotic Resistance Genes

Mobilized colistin resistance genes (*mcr-1* to *mcr-9*) among all *E. coli* isolates in this study were identified using multiplex PCRs as previously described [30,31]. Carbapenemase-encoding genes including *bla*_IMP_, *bla*_VIM_, *bla*_NDM_, *bla*_SPM_, *bla*_AIM_, *bla*_DIM_, *bla*_GIM_, *bla*_SIM_, *bla*_KPC_, *bla*_BIC_ and *bla*_OXA−48_ were detected by PCRs [32]. Plasmid-mediated tigecycline resistance determinant *tet*(X4) was amplified as previously described [33]. All *E. coli* isolates were also screened for the presence of plasmid-mediated quinolone resistance (PMQR) genes (*qnrA*, *qnrB*, *qnrS*, *qnrC*, *qnrD*, *qepA*, and *oqxAB*) [34,35,36,37,38]. The presence of extended-spectrum β-lactamases (ESBLs) (*bla*_CTX-M-1G_, *bla*_CTX-M-9G_, *bla*_CTX-M-2G_, and *bla*_CTX-M-25G_) and plasmid-mediated AmpC β-lactamases (pAmpC) (*bla*_CMY-2_ and *bla*_DHA-1_) both conferring resistance to cephalosporins was also analyzed as previously described [39,40,41,42]. Resistance genes *rmtB*, *fosA* and *fosA3* were also screened as previously reported [43,44].

### 2.4. Detection and Serotyping of ExPEC Isolates

All isolates were investigated for the following five key virulence markers: *papA* and/or *papC* (P fimbriae; counted as 1), *sfa* and/or *foc* (S and F1C fimbriae, respectively), *afa* and/or *dra* (Afimbrial and Dr-binding adhesion, respectively), *kpsM* II (group 2 capsule), and *iutA* (aerobactin system). The isolates carrying ≥ 2 of the above 5 ExPEC-defining markers were classified as ExPEC [6]. All PCR amplicons were sequenced to confirm these virulence genes.

After PCR identification of ExPEC, the 30 most prevalent serogroups including O1, O2, O4, O6, O7, O8, O9, O15, O18, O21, O22, O25, O26, O45, O55, O78, O83, O86, O101, O103, O111, O113, O117, O121, O138, O145, O149, O157, O158 and O165, were screened among these ExPEC isolates by PCRs as previously described [45].

### 2.5. Pulse-Field Gel Electrophoresis (PFGE) and Multilocus Sequence Typing (MLST)

To determine the genetic relationship between ExPEC isolates, PFGE was carried out as previously described [46]. Briefly, the ExPEC isolates were grown on Luria–Bertani agar (Qingdao Haibo Microorganism Reagent Co., Ltd., Qingdao, China) overnight at 37 °C and diluted to an optical density of 0.5. Subsequently, the bacteria dilutions were embedded in SeaKem Gold agarose (Lonza, Rockland, ME, United States) and culture plugs were lysed with 100 μg mL^−1^ protease K (Solarbio, Beijing, China) by incubation in a shaking water bath at 55 °C for 2 h. Then, the lysed plugs were washed using sterilized water and Tris–EDTA buffer, respectively. The plugs were then digested with *Xba*I (TaKaRa, Dalian, China) and subjected to PFGE analysis using Chef Mapper electrophoresis system (Bio-Rad Laboratories). The gels were run at 6.0 V cm^−1^ with an initial/final switch time of 2.16 s/54.17 s for 19 h. PFGE patterns were analyzed with BioNumerics software version 7.0 (Applied Maths, Kortrijk, Belgium) by using Dice coefficients and the unweighted-pair group method to achieve dendrograms with a 1.5% band position tolerance. *Salmonella enterica* serotype Braenderup H9812 standards served as size markers.

The ExPEC isolates were also subtyped by the multilocus sequence typing (MLST) method using seven house-keeping genes (*adk*, *fumC*, *gyrB*, *icd*, *mdh*, *recA* and *purA*) of *E. coli* as previously described [47]. All the PCR amplicons were sequenced and imported into the *E. coli* MLST database website (https://pubmlst.org/bigsdb?db=pubmlst_escherichia_seqdef&page=sequenceQuery (accessed on 29 July 2020)).

### 2.6. Statistical Analysis

Differences in proportions were compared using the χ^2^ test implemented in SPSS software (Version 17.0; SPSS Inc., Chicago, IL, USA). All tests of significance were two-tailed, and a value of *p* *≤* 0.05 was considered statistically significant.

## 3. Results

### 3.1. Antimicrobial Susceptibilities

A total of 926 *E. coli* isolates were obtained including 78 isolates from white-feather broilers, 371 isolates from brown-egg layers and 477 isolates from yellow-feather broilers in 17 live poultry markets (Table 1). As shown in Table 1, resistances to tetracyclines were observed most often among the total 926 *E. coli* isolates in this study, and 89.3% and 83.8% of the isolates were resistant to tetracycline and doxycycline, respectively, although none of the isolates were resistant to the newly tetracyclines drug, tigecycline, a last-resort treatment for infections caused by MDR Gram-negative bacteria in humans. For the β-lactam drugs, the rate of resistance to ampicillin was the highest (87.1%), followed by ceftiofur (44.7%), cefotaxime (41.8%), and meropenem (4.9%). Among aminoglycosides, resistance to streptomycin was the greatest (60.7%), followed by kanamycin (50.5%), gentamicin (31.9%), and amikacin (8.9%). For quinolones, the old quinolone drug nalidixic acid possessed the highest resistance rate (77.1%), and the rates of resistance to the three fluoroquinolones (enrofloxacin, ciprofloxacin, and levofloxacin) varied from 36.3% to 58.4%. Moreover, 20.6% and 69.1% of these isolates were resistant to fosfomycin and florfenicol, respectively. Worryingly, 17.0% of the isolates were resistant to colistin, a critically important antimicrobial for humans (Table 1).

For isolates from white-feather broilers, brown-egg layers and yellow-feather broilers (live poultry markets), respectively, the rates of resistance to ampicillin, tetracycline and doxycycline were all above 80.0% (Table 1). Notably, except levofloxacin and tigecycline, the resistance rate for each of the remaining 15 antimicrobials tested among *E. coli* from white-feather broilers, was significantly higher than that from brown-egg layers and that from yellow-feather broilers (*p* < 0.05) (Table 1). The rate of resistance to ampicillin, cefotaxime, colistin, and fosfomycin among *E. coli* from yellow-feather broilers was significantly higher than that from brown-egg layers, respectively (*p* < 0.05). For meropenem, levofloxacin and streptomycin, respectively; however, the *E. coli* isolates from brown-egg layers possessed significantly higher resistance rate than that from yellow-feather broilers (*p* < 0.05) (Table 1).

### 3.2. Detection of Resistance Genes

Among the 926 *E. coli* isolates, *bla*_NDM_ was found in 45 (4.9%) isolates, and no other carbapenemase-encoding genes was found in this study (Table 2). For the ESBLs-encoding genes, *bla*_CTX-M-9G_ found in 222 (24.0%) of the total *E. coli* isolates was the most prevalent gene, consisting of 52 (14.0%) from 371 brown-egg layers, 54 (69.2%) from 78 white-feather broilers and 116 (24.3%) from 477 yellow-feather broilers of live poultry markets. There were 130 isolates (14.0%) carrying *bla*_CTX-M-1G_, including 43, 32 and 55 isolates from layer farms, broiler farm and live poultry markets, respectively. A total of 22 isolates harbored both *bla*_CTX-M-1G_ and *bla*_CTX-M-9G_ and no other ESBLs-encoding genes was found in this study. pAmpC-encoding genes *bla*_CMY-2_ and *bla*_DHA-1_ were found in 53 (5.7%) and 3 (0.3%) of the 926 isolates. Among the PMQR determinants, *qnrS* and *oqxAB* found in 311 (33.6%) and 181 (19.5%) isolates, respectively, were the two most prevalent genes, followed by *qnrB* (34, 3.7%) and *qnrD* (21, 2.3%) (Table 2). There was no *qnrA* and *qnrC* found in this study. Of the 926 *E. coli* isolates, plasmid-borne fosfomycin resistance (PFR) genes were found in 191 isolates, and the number of isolates harboring *fosA3* and *fosA* was 189 and 2, respectively. Notably, 157 (17.0%) of the isolates were found to harbor *mcr-1*, consisting of 22 (5.9%) from 371 brown-egg layers, 53 (67.9%) from 78 white-feather broilers and 82 (17.2%) from 477 yellow-feather broilers, and no other *mcr* genes were found in this study. In addition, *rmtB* was present in 35 isolates (2.8%). Luckily, none of the 926 isolates carried the plasmid-mediated tigecycline-resistance determinant *tet*(X4).

The detection rate of *bla*_NDM_, *bla*_CTX-M-9G_, *bla*_CTX-M-1G_, *mcr-1*, *qnrS*, *fosA3* and *rmtB* in *E. coli* from white-feather broilers, respectively, was significantly higher than that from layer farms and that from live poultry markets (*p* < 0.05) (Table 2). For *bla*_CTX-M-9G_, *bla*_CMY-2_, *mcr-1*, *oqxAB*, *qnrB*, *qnrD*, *fosA3* and *rmtB*, respectively, the yellow-feather broilers from live poultry markets possessed significantly higher detection rate than that from brown-egg layers (*p* < 0.05) (Table 2).

### 3.3. Prevalence of ExPEC Isolates and Their Serogroups

**Figure 1:**
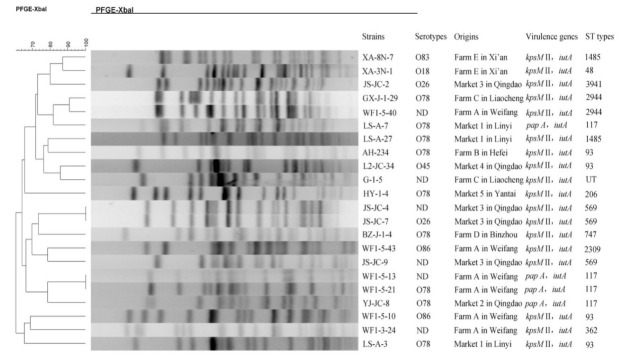
Characteristics and PFGE dendrogram patterns of the 22 ExPEC isolates from healthy chickens in this study.

In the present study, 22 (2.4%) of the 926 chicken isolates were qualified as ExPEC. As shown in Table 3, six ExPEC isolates were found in the white-feather broiler farm, and a total of six ExPEC isolates were also detected in four of the seven layer farms. The detection rates of ExPEC ranged from 0.7% to 6.3% among the four layer farms. Chickens harboring ExPEC in live poultry markets were found in three of the six cities we collected samples from and the detection rates of ExPEC among the yellow-feather broilers from poultry markets were 4.4% (3/68) in city Linyi, 3.0% (6/199) in city Qingdao and 1.8% (1/55) in city Yantai (Table 3). The six ExPEC isolates in Qingdao were from three live poultry markets (Table 3 and Figure 1). Notably, the detection rate (7.7%, 6/78) of ExPEC among white-feather broilers was significantly higher than that (1.6%, 6/371) from brown-egg layers and that (2.1%, 10/477) from yellow-feather broilers (*p* < 0.05). There was no significant difference between the detection rate (1.6%, 6/371) of ExPEC among brown-egg layers and that among yellow-feather broilers (2.1%, 10/477) (*p* = 0.611) (Table 3).

Among the 22 ExPEC isolates, *iutA* was the most prevalent ExPEC-defining marker, followed by *kpsM* II (18 isolates) and *papA* (4 isolates) (Figure 1). All ExPEC isolates carried two of the five ExPEC-defining markers, and no other markers were found in our study. After the PCR-based serotyping method was applied to the 22 ExPEC isolates, the serogroups of 16 isolates were successfully identified and they belonged to six serogroups (O78, O26, O86, O18, O45 and O83). O78 detected in nine of the 22 ExPEC isolates (40.9%) was the most prevalent serogroup, followed by O26 (9.1%, 2/22) and O86 (9.1%, 2/22) (Figure 1).

### 3.4. Antimicrobial Resistance Phenotypes and Genotypes of the ExPEC Isolates

**Figure 2:**
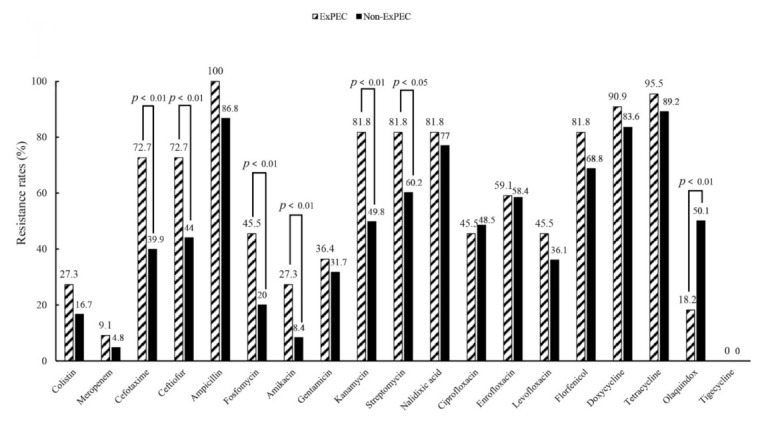
Comparison of the antibiotic resistance rates between the ExPEC and non-ExPEC isolates in this study.

An antimicrobial susceptibility test showed that all 22 ExPEC isolates were resistant to ampicillin (100%) and sulfamethoxazole-trimethoprim (100%), followed by resistance to tetracycline (95.5%), and doxycycline (90.9%) (Table 4 and Figure 2). All the rates of resistance to florfenicol, streptomycin, kanamycin and nalidixic acid among these isolates were 81.8%. For the third-generation cephalosporins, the rates of resistance to cefotaxime and ceftiofur were both 72.7% (16/22), and the resistance rates to fluoroquinolones ranged from 45.5% to 59.1% (Figure 2). The number of isolates resistant to amikacin and fosfomycin were six (27.3%) and ten (45.5%), respectively. A total of six (27.3%) and two (9.1%) ExPEC isolates were resistant to the two critically important antibiotics colistin and meropenem, respectively. Notably, two ExPEC isolates WF1-5-13 and WF1-5-40 were resistant to both colistin and meropenem (Table 4). Luckily, no isolate was resistant to tigecycline. Detailed results of the antibiotic resistance profiles for the 22 ExPEC isolates were presented in Table 4. Interestingly, the rate of resistance to cefotaxime, ceftiofur, fosfomycin, amikacin, kanamycin and streptomycin in ExPEC isolates was significantly higher than that of non-ExPEC isolates in this study, respectively (*p* < 0.05) (Figure 2). Worryingly, all the ExPEC isolates in this study were MDR (resistance ≥ 3 three classes of antibiotics) in nature (Table 4).

Among the 16 ExPEC isolates resistant to cefotaxime, *bla*_CTX-M_ was found in 15 isolates (10 *bla*_CTX-M-9G_ and 6 *bla*_CTX-M-1G_), including one isolate WF1-5-13 carrying both *bla*_CTX-M-9G_ and *bla*_CTX-M-1G_ (Table 4). *bla*_CTX-M-9G_ and *bla*_CMY-2_-type pAmpC-encoding gene were present in isolate AH234 from layer farm B, accounting for the resistances to the third-generation cephalosporins. *fosA3* was found in ten ExPEC isolates resistant to fosfomycin and this gene was only distributed among *bla*_CTX-M_-positive isolates. For the six colistin and two meropenem resistant ExPEC isolates, the presence of *mcr-1* and *bla*_NDM_ could account for their corresponding resistance, respectively. Notably, the two ExPEC isolates (WF1-5-13 and WF1-5-40) harboring both *mcr-1* and *bla*_NDM_, carried additional *bla*_CTX-M_, *fosA3* and *floR* genes, and isolate WF1-5-40 also possessed *rmtB* (Table 4).

### 3.5. Genetic Relationships of the ExPEC Isolates

All 22 ExPEC isolates could be successfully analyzed by PFGE and 20 different PFGE profiles were obtained indicating that clonal dissemination of these ExPEC isolates was unlikely (Figure 1). As shown in Figure 1, 12 types were identified by the MLST subtyping method along with one new ST (6-6-804-10-9-1-6 in isolate G-1-5) not previously registered in the *E. coli* MLST database. The most prevalent ST types were ST117 (four) and ST93 (four), followed by ST569 (three), ST1485 (two) and ST2944 (two). Most isolates sharing the same ST types had different PFGE profiles and were from different farms or markets of different cities (Figure 1). For example, the four isolates (AH234, L2-JC-34, WF1-5-10 and LS-A-3) belonging to ST93 were from four chicken markets/farms in four cities and had different PFGE patterns. Notably, the two ExPEC isolates WF1-5-13 and WF1-5-21 from the same chicken farm in city Weifang shared identical PFGE pattern and ST type; however, different resistance genotypes and phenotypes were found in these two isolates (Figure 1 and Table 4). Different resistance phenotypes were also found in isolates JS-JC-4 and JS-JC-7, which were from the same market and possessed identical PFGE pattern and ST type (Figure 1 and Table 4).

## 4. Discussion

The presence of ExPEC colonizing healthy chickens could be a huge threat to both animal and human health. For China, having the largest consumption of antibiotics in the world, the prevalence and antibiotic resistance of ExPEC among healthy chickens urgently need to be studied. In this study, we investigated the resistance of *E. coli* isolates from healthy chickens of seven layer farms, one white-feather broiler farm and 17 live poultry markets in China, and the ExPEC among these commensal isolates were also characterized.

Both the rates of resistance to tetracycline and doxycycline among isolates in this study were above 80.0%, consistent with that from chickens in China after 2012 [48]. The high resistance rates to these drugs could be attributed to the heavy usage of tetracyclines in poultry, because oxytetracycline, tetracycline, chlortetracycline and doxycycline have been heavily used for decades in animal production including poultry [49]. Tigecycline, a last-resort treatment for human infections caused by MDR Gram-negative bacteria, has never been used in animal husbandry. Luckily, isolate resistant to tigecycline was not found in our *E. coli* isolated during 2015–2017; however, the heavy usage of tetracyclines in animals could increase the prevalence of newly mobile tigecycline-resistance gene *tet*(X4) in *E. coli* and this should be paid more attention [33]. The resistance rates to meropenem (4.9%) and colistin (17.0%), two critically important antimicrobials in human medicine, among the 926 isolates in our study could be well accounted for the presence of *bla*_NDM_ (4.9%) and *mcr-1* (17.0%), respectively (Table 1 and Table 2). *qnrS* was the most prevalent PMQR gene in this study, differing from that in humans [50] and animals [51], in which the most prevalent PMQR gene was *oqxAB*. In some countries, *qnrB* was the most prevalent type [52]. The prevalence of CTX-M-type ESBLs (35.6%) in healthy chickens in this study was similar to that (38.5%) in *E. coli* isolates from chickens in China [48], but lower than that in chicken production of India [53]. Notably, except levofloxacin and tigecycline, the resistance rate for each of the 15 antimicrobials tested among *E. coli* from white-feather broilers, was significantly higher than that from brown-egg layers and that from yellow-feather broilers (*p* < 0.05) (Table 1). Such phenomena could be attributed to that consumption of antibiotics in white-feather broilers is the largest among the three types of chickens. The rate of resistance to ampicillin, cefotaxime, colistin, and fosfomycin among *E. coli* from yellow-feather broilers was significantly higher than that from brown-egg layers, respectively (*p* < 0.05). This might because that β-lactams and colistin are often used in early feeding period of the yellow-feather broilers while almost all antimicrobials are forbidden in layer farms during the laying period.

Based on the molecular criteria of Johnson et al. [6], 2.4% (22/926) of the healthy chicken fecal *E. coli* isolates were qualified as ExPEC in this study. The ExPEC could asymptomatically colonize the gut of a fraction of healthy animal population and survive in extra-intestinal environments, causing diseases in animals and humans through the food chain [54]. The threat to human health posed by the healthy chicken ExPEC isolates in this study could been further proved by that healthy poultry ExPEC were capable to adhere or invade human intestinal epithelial [14]. In this study, virulence markers *iutA* and *KpsM* II were the two most prevalent genes among the ExPEC isolates from healthy chickens, consistent with the finding about MDR *E. coli* in healthy chickens in Brazil [14]. Besides isolation methods, geographic locations and management practices, different classification criteria for ExPEC has been the main factor contributing to differences in frequency of ExPEC between studies. We will focus the studies using the same PCR-based screening method for ExPEC as that in our study. The detection rate of ExPEC in our samples was 2.4%, similar to that (4.7%, 5/108) among chicken egg *E. coli* isolates (*p* > 0.05), but lower than that (21.5%, 130/606) in chicken meat isolates reported in the USA (*p* < 0.05) [55]. Notably, the prevalence of ExPEC isolates (2.4%, 22/926) in our study was also significantly lower than that (13.2%, 40/304) from farmed healthy chickens in Quebec, Canada [20]. This might be explained by that boiled DNA extracts from total cultures of samples were initially screened for all possible ExPEC strains in the previous study, contributing to a high recovery rate of ExPEC. In this study, the detection rate (7.7%, 6/78) of ExPEC among white-feather broilers was significantly higher than that (1.6%, 6/371) from brown-egg layers and that (2.1%, 10/477) from yellow-feather broilers in live poultry markets (*p* < 0.05). The phenomenon could be attributed to the selective pressure on the dissemination of ExPEC posed by antimicrobials frequently used in white-feather broilers. This was proved by that highly similar PFGE patterns were found in the two ExPEC isolates WF1-5-13 and WF1-5-21 from the same Farm (Figure 1).

In this study, the most two prevalent ST types of the 22 ExPEC isolates were ST117 and ST93 (Figure 1). Since ST117 and ST93 types of ExPEC had been found to be associated with meningitis of humans in Brazil in 1999 (http://enterobase.warwick.ac.uk/species/ecoli/search_strains?query=st_search (accessed on 7 October 2020)), both ST types of *E. coli* have caused sepsis among humans [56,57] and disseminated among humans around the world including the European countries and China (http://enterobase.warwick.ac.uk/species/ecoli/search_strains?query=st_search (accessed on 7 October 2020)). Besides ST2944, all other STs in this study have been also found in human isolates (http://enterobase.warwick.ac.uk/species/ecoli/search_strains?query=st_search (accessed on 7 October 2020)). Serogroups of the ExPEC isolates from animals were rarely studied, although the APEC isolates from diseased poultry were often serotyped. In this study, O78 was the most predominant serogroup among ExPEC isolates from healthy chickens, followed by O26 and O86. This was slightly different from that of a previous study about APEC from Korea in which O78 was the most prevalent serogroup followed by O2 and O53 [15], both of which were not identified in our study. Notably, O78 was also a common serogroup among human ExPEC isolates from neonatal meningitis in Europe [58]. The distribution rate of each serogroup in this study was also different from that of APEC obtained between 2005 and 2008 in Guangdong, China [59]. The presence of serogroup O86 in two ExPEC from healthy chickens in this study are of interest since this serogroup was only identified in human ExPEC strains in Brazil rather than in strains from poultry in a previous report [57]. In the present study, one ExPEC isolate from healthy chicken belonged to O18 serogroup, which was frequently found in APEC from diseased avian in the United States and also the main serogroup of ExPEC isolates causing newborn meningitis in the Europe [58,60,61]. O83 and O45, which were prevalent in neonates with *E. coli* meningitis from the Netherlands [61] and France [62], respectively, were also found in our ExPEC isolates. All these results show that the ExPEC isolates from healthy chickens in this study might transmit to humans and the prevalence of ExPEC isolates in healthy animals should be monitored in the future.

Notably, all the ExPEC isolates in this study were MDR isolates. Although the use of antibiotics in animals does not select ExPEC strains intrinsically [25], it will favor the dissemination of ExPEC with MDR phenotypes among healthy animals, contributing to the emergence of MDR ExPEC in human infections [22]. Almost all ExPEC isolates in this study were resistant to ampicillin (100%), sulfamethoxazole-trimethoprim (100%), tetracycline (95.5%), and doxycycline (90.9%), which were all used extensively in animal husbandry in China, further favoring the dissemination of ExPEC among animals and humans by co-selection. In recent years, ExPEC isolates producing ESBLs or AmpC in human infections have been increasing [63], and such pathogens have been also found in healthy poultry in Brazil recently [14]. In our study, CTX-M-type ESBLs were found in 15 of the 22 ExPEC isolates from healthy chickens. The increase in ESBLs or AmpC among ExPEC from poultry will inevitably reduce the therapeutic options of ExPEC infections in humans, because cephalosporins are important to human medicine. Fosfomycin has been widely recommended for treating uncomplicated urinary tract infection especially caused by ESBLs-producing or fluoroquinolone-resistant ExPEC isolates [64]. However, ten ExPEC isolates in this study harbored the fosfomycin resistance gene *fosA3* and all ten isolates also carried *bla*_CTX-M_, among which six were resistant to fluoroquinolones (Table 4). Such pathogens in healthy chickens will pose a great threat to human health because they will compromise the efficacies of fosfomycin, fluoroquinolones and cephalosporins. For ExPEC from different markets/farms, three isolates (YJ-JC-8, WF1-5-21 and LS-A-7) carrying virulence genes *papA* and *iutA* belonged to serogroup O78 and they were all ST117 type, however, different PFGE and resistance profiles were present in the three isolates (Figure 1 and Table 4). This indicates that the ExPEC isolates have undergone a complex evolutionary process resulting in genetically diverse isolates although they share identical ST type and serogroup at the beginning. Even for ExPEC isolates with identical PFGE pattern and ST type from the same farm, such as WF1-5-13 and WF1-5-21, different resistance genotypes and phenotypes were also observed, further proving the complex evolutionary process within ExPEC.

Worryingly, besides *mcr-1* and *bla*_NDM_, the two ExPEC isolates WF1-5-13 and WF1-5-40 carried additional *bla*_CTX-M_, *fosA3* and *floR* genes, with isolate WF1-5-40 also harboring *rmtB* (Table 4). The presence of such ExPEC isolates co-harboring *bla*_NDM_ and *mcr-1* in healthy chickens in this study will threaten the health of consumers because such pathogens will not only compromise the efficacies of cephalosporins, fosfomycin and aminoglycosides, but also threaten the usage of carbapenems and colistin, two critically important antimicrobials used for serious infections caused by MDR ExPEC [65]. *mcr* has been also found in two ExPEC isolates from diseased poultry in Brazil [18] and two ExPEC isolates from healthy ducks in China [66]. All four ExPEC isolates carrying *mcr* from animals in the two previous reports were susceptible to carbapenems, although NDM-producing ExPEC isolates susceptible to colistin have been reported in humans [67]. To the best of our knowledge, this is the first report about *mcr-1*-positive ExPEC isolates harboring *bla*_NDM_ from healthy chickens.

## 5. Conclusions

In conclusion, we observed that the resistances in *E. coli* from white-feather broilers were more serious than those from layer farms and those from live-poultry markets in China, respectively. This study also reported that 2.4% of these *E. coli* isolates from healthy chickens were qualified as ExPEC using a molecular detection method. The most predominant serogroup of these ExPEC isolates was O78, followed by O26 and O86, and almost all serogroups identified in our study were frequently reported in human ExPEC isolates in many countries, suggesting that ExPEC isolates from healthy poultry could be a source of potentially virulent ExPEC causing multiple diseases in humans. Notably, all the ExPEC isolates in this study possessed MDR phenotypes and most showed resistances to cephalosporins and fosfomycin, which made co-selection of these ExPEC possible when corresponding drugs were used. More worryingly, six ExPEC isolates in this study carried *mcr-1*, including two harboring both *bla*_NDM_ and *mcr-1*, which could compromise both the efficacies of carbapenems and colistin. The presence of MDR ExPEC isolates in healthy chickens, especially those carrying *mcr-1* and/or *bla*_NDM_, is alarming and will pose a serious health threat to consumers. Interventions need to be taken to reduce these pathogens in the chicken intestine and prevent clinical ExPEC infections in humans by reducing transmission via poultry products. Further studies are required for monitoring the prevalence of MDR ExPEC in healthy chickens in China and other countries. To our knowledge, this is the first report of *mcr-1*-positive ExPEC isolates harboring *bla*_NDM_ from healthy chickens.

## Figures and Tables

**Table 1:** Comparison of the resistance rates of *E. coli* isolates from chickens of different origins.

Antimicrobials	Resistance Rates of Isolates (%) *			
	Total (*n* = 926)	Layer Farms (*n* = 371)	White-Feather Broiler Farms (*n* = 78)	Live Poultry Markets (*n* = 477)
β-lactams	-	-	-	-
AMP	87.1	83.0 ^a^	100 ^b^	88.3 ^c^
CTF	44.7	39.4 ^a^	98.7 ^b^	40.0 ^a^
CTX	41.8	26.1 ^a^	100 ^b^	41.5 ^c^
MEM	4.9	1.9 ^a^	48.7 ^b^	0.0 ^c^
Quinolones	-	-	-	-
NAL	77.1	77.6 ^a^	96.2 ^b^	73.6 ^a^
ENR	58.4	59.3 ^a^	89.7 ^b^	52.6 ^a^
CIP	48.4	45.8 ^a^	83.3 ^b^	44.7 ^a^
LEV	36.3	40.2 ^a^	51.3 ^a^	30.8 ^b^
Tetracyclines	-	-	-	-
TET	89.3	87.3 ^a^	97.4 ^b^	89.5 ^a^
DOX	83.8	81.1 ^a^	97.4 ^b^	83.6 ^a^
TIG	0	0	0	0
Aminoglycosides	-	-	-	-
STR	60.7	63.9 ^a^	83.3 ^b^	54.5 ^c^
KAN	50.5	46.6 ^a^	96.2 ^b^	46.1 ^a^
GEN	31.9	26.1 ^a^	73.1 ^b^	29.6 ^a^
AMK	8.9	5.1 ^a^	35.9 ^b^	7.3 ^a^
Polypeptides	-	-	-	-
COL	17.0	4.9 ^a^	73.1 ^b^	17.2 ^c^
Others	-	-	-	-
FFC	69.1	68.7 ^a^	94.9 ^b^	65.2 ^a^
FOS	20.6	10.5 ^a^	78.2 ^b^	19.1 ^c^

* The different lowercase letters in the same line were considered significantly different (*p* ≤ 0.05) between two groups using a χ2 test with SPSS software version 19.0. OLA, olaquindox; COL, colistin; FFC, florfenicol; DOX, doxycycline; AMP, ampicillin; CTX, cefotaxime; CTF, ceftiofur; CIP, ciprofloxacin; LEV, levofloxacin; FOS, fosfomycin; MEM, meropenem; NAL, nalidixic acid; GEN, gentamicin; ENR, enrofloxacin; KAN, kanamycin; STR, streptomycin; AMK, amikacin; TET, tetracycline. TIG, tigecycline.

**Table 2:** Prevalence of resistance genes among the 926 *E. coli* isolates from chickens.

Resistance Genes	No. of Positive Isolates (%) *			
	Total (*n* = 926)	Layer Farms (*n* = 371)	White-Feather Broiler Farms 9 (*n* = 78)	Live Poultry Markets (*n* = 477)
Carbapenemases	-	-	-	-
*bla*_NDM_	45 (4.9)	11 (3.0) ^a^	34 (43.6) ^b^	0 (0) ^c^
ESBLs	-	-	-	-
*bla*_CTX-M-9G_	222 (24.0)	52 (14.0) ^a^	54 (69.2) ^b^	116 (24.3) ^c^
*bla*_CTX-M-1G_	130 (14.0)	43 (11.6) ^a^	32 (41.0) ^b^	55 (11.5) ^a^
pAmpC	-	-	-	-
*bla*_CMY-2_	53 (5.7)	11 (3.0) ^a^	3 (3.8) ^ab^	39 (8.2) ^b^
*bla*_DHA-1_	3 (0.3)	1 (0.3) ^a^	0 (0.0) ^a^	2 (0.4) ^a^
MCR	-	-	-	-
*mcr-1*	157 (17.0)	22 (5.9) ^a^	53 (67.9) ^b^	82 (17.2) ^c^
PMQR	-	-	-	-
*oqxAB*	181 (19.5)	41 (11.1) ^a^	22 (28.2) ^b^	118 (24.7) ^b^
*qnrB* *	34 (3.7)	7 (1.9) ^a^	1 (1.3) ^ab^	26 (5.5) ^b^
*qnrS*	311 (33.6)	140 (37.7) ^a^	12 (15.4) ^b^	159 (33.3) ^a^
*qnrD* *	21 (2.3)	3 (0.8) ^a^	0 (0.0) ^ab^	18 (3.8) ^b^
PFR	-	-	-	-
*fosA3*	189 (20.4)	39 (10.5) ^a^	61 (78.2) ^b^	89 (18.7) ^c^
*fosA*	2 (0.2)	0 (0.0) ^a^	0 (0.0) ^a^	2 (0.4) ^a^
Others	-	-	-	-
*rmtB*	35 (3.8)	2 (0.5) ^a^	19 (24.4) ^b^	14 (2.9) ^c^

* The different lowercase letters in the same line were considered significantly different (*p* ≤ 0.05) between two groups using a χ2 test with SPSS software version 19.0.

**Table 3:** Prevalence and origins of the 22 ExPEC isolates in this study.

Location	Origins	No. of Farms/Markets	Year	ExPEC Isolates (%)/ Total Isolates
Weifang in Shandong	White-feather broiler farm	1	2015	6 (7.7%)/78
Hefei in Anhui	Layer farm	1	2015	1 (6.3%)/16
Liaocheng in Shandong	Layer farm	1	2015	2 (4.9%)/41
Binzhou in Shandong	Layer farms	2	2016	1 (0.7%)/135
Xi’an in Shanxi	Layer farm	1	2015	2 (2.1%)/97
Qingdao in Shandong	Layer farms	2	2017	0/82
Linyi in Shandong	Live poultry market	1	2015	3 (4.4%)/68
Qingdao in Shandong	Live poultry markets	11	2015	6 (3.0%)/199
Yantai in Shandong	Live poultry markets	2	2015	1 (1.8%)/55
Zaozhuang in Shandong	Live poultry market	1	2015	0/57
Zibo in Shandong	Live poultry market	1	2015	0/67
Weifang in Shandong	Live poultry market	1	2015	0/31
-	White-feather broiler farm	1	-	6 (7.7%)/78
-	Layer farms	7	-	6 (1.6%)/371
-	Live poultry markets	17	-	10 (2.1%)/477

**Table 4:** Resistance phenotypes and genotypes of the 22 ExPEC isolates in this study.

Strain (ST Types)	Source	City	Serotype #	ExPEC-Defining Markers	Resistance Phenotype	Resistance Genes
WF1-3-24	White-feather broiler farm A	Weifang	ND	*KpsM II, iutA*	*mcr-1*, *bla*_CTX-M-1G_, *fosA3*, *rmtB*, *oqxAB*, *floR*	COL, CTX, CTF, AMP, FOS, AMK, GEN, KAN, STR, NAL, CIP, ENR, LEV, FFL, DOX, TET, OLA, SXT
WF1-5-10	White-feather broiler farm A	Weifang	O86	*KpsM II, iutA*	*bla*_CTX-M-9G_, *fosA3*, *rmtB*, *floR*	CTX, CTF, AMP, AMK, GEN, KAN, STR, NAL, CIP, ENR, LEV, FFL, DOX, TET, SXT
WF1-5-13	White-feather broiler farm A	Weifang	ND	*papA, iutA*	*mcr-1*, *bla*_NDM-5_, *bla*_CTX-M-9G_, *bla*_CTX-M-1G_, *fosA3*, *floR*	COL, MEM, CTX, CTF, AMP, FOS, KAN, STR, NAL, CIP, ENR, LEV, FFL, DOX, TET, SXT
WF1-5-21	White-feather broiler farm A	Weifang	O78	*papA, iutA*	*mcr-1*, *bla*_CTX-M-9G_, *fosA3, rmtB*, *floR*	COL, CTX, CTF, AMP, FOS, AMK, GEN, KAN, STR, NAL, FFL, DOX, TET, SXT
WF1-5-40	White-feather broiler farm A	Weifang	ND	*KpsM II, iutA*	*mcr-1*, *bla*_NDM-1_, *bla*_CTX-M-9G_, *fosA3*, *rmtB*, *floR*	COL, MEM, CTX, CTF, AMP, FOS, AMK, GEN, KAN, STR, FFL, DOX, TET, SXT
WF1-5-43	White-feather broiler farm A	Weifang	O86	*KpsM II, iutA*	*bla*_CTX-M-9G_, *fosA3*	CTX, CTF, AMP, FOS, KAN, STR, NAL, CIP, ENR, LEV, DOX, TET, SXT
AH-234	Layer farm B	Hefei	O78	*KpsM II, iutA*	*bla*_CTX-M-9G_, *bla*_CMY-2_, *floR*	CTX, CTF, AMP, AMK, GEN, KAN, STR, NAL, CIP, ENR, LEV *, FFL, TET, SXT
G-1-5	Layer farm C	Liaocheng	ND	*KpsM II, iutA*	*mcr-1*	COL, AMP, KAN, STR, NAL, DOX, TET, SXT
GX-J-1-29	Layer farm C	Liaocheng	O78	*KpsM II, iutA*	*qnrS*, *floR*	AMP, FFL, DOX, TET, SXT
BZ-J-1-4	Layer farm D	Binzhou	O78	*KpsM II, iutA*	*bla*_CTX-M-1G_, *fosA3*	CTX, CTF, AMP, FOS, STR, NAL, FFL, DOX, TET, SXT
XA-8N-7	Layer farm E	Xi’an	O83	*KpsM II, iutA*	*bla*_CTX-M-1G_, *floR*	CTX, CTF, AMP, KAN, NAL, ENR, LEV, FFL, DOX, TET, SXT
XA-3N-1	Layer farm E	Xi’an	O18	*KpsM II, iutA*	*bla*_CTX-M-1G_, *floR*	CTX, CTF, AMP, KAN, STR, NAL, CIP, ENR, LEV, FFL, DOX, TET, SXT
LS-A-3	Live poultry market 1	Linyi	O78	*KpsM II, iutA*	*bla*_CTX-M-9G_, *fosA3*, *floR*	CTX, CTF, AMP, FOS, KAN, STR, NAL, CIP, ENR, LEV, FFL, DOX, TET, SXT
LS-A-7	Live poultry market 1	Linyi	O78	*papA, iutA*	*bla*_CTX-M-1G_, *fosA3*, *oqxAB*, *floR*	CTX, CTF, AMP, FOS, KAN, STR, NAL, CIP, ENR, LEV, FFL, DOX, TET, OLA, SXT
LS-A-27	Live poultry market 1	Linyi	O78	*KpsM II, iutA*	*bla*_CTX-M-9G_, *floR*	CTX, CTF, AMP, KAN, STR, NAL, ENR, FFL, DOX, TET, SXT
YJ-JC-8	Live poultry market 2	Qingdao	O78	*papA, iutA*	*oqxAB*	AMP, AMK, GEN, KAN, STR, NAL, CIP, ENR, LEV, FFL, DOX, TET, OLA, SXT
JS-JC-2	Live poultry market 3	Qingdao	O26	*KpsM II, iutA*	*floR*	CTX, CTF, AMP, FOS, GEN, KAN, STR, NAL, CIP, ENR, LEV, FFL, DOX, TET, SXT
JS-JC-4	Live poultry market 3	Qingdao	ND	*KpsM II, iutA*	*qnrS*	AMP, KAN, STR, NAL, DOX, TET, SXT
JS-JC-7	Live poultry market 3	Qingdao	O26	*KpsM II, iutA*	*qnrS*	AMP, STR, DOX, TET, SXT
JS-JC-9	Live poultry market 3	Qingdao	ND	*KpsM II, iutA*	*bla*_CTX-M-9G_, *qnrS*	CTX, CTF, AMP, KAN, STR, FFL, DOX, TET, SXT
L2-JC-34	Live poultry market 4	Qingdao	O45	*KpsM II, iutA*	*mcr-1*, *bla*_CTX-M-9G_, *fosA3*, *floR*	COL, CTX, CTF, AMP, FOS, GEN, KAN, NAL, FFL, SXT
HY-1-4	Live poultry market 5	Yantai	O78	*KpsM II, iutA*	-	AMP, NAL, ENR, FFL, DOX, TET, SXT

# ND, not determined; * Intermediate resistance; AMP, ampicillin; MEM, meropenem; CTX, cefotaxime; CTF, ceftiofur; CAZ, Ceftazidime; ETP, ertapenem; IPM, imipenem; NAL, nalidixic acid; CIP, ciprofloxacin; ENR, enrofloxacin; LEV, levofloxacin; STR, streptomycin; KAN, kanamycin; GEN, gentamicin; AMK, amikacin; TET, tetracycline; DOX, doxycycline; FOS, fosfomycin; FFC, florfenicol; TIG, tigecycline; SXT, sulfamethoxazole-trimethoprim (SMZ-TMP).

## Data Availability

No new data were created or analyzed in this study. Data sharing is not applicable to this article.

## Supplementary Materials

The following are available online at https://www.mdpi.com/article/10.3390/ani11041112/s1, Table S1: Origins of the samples collected in this study.
