# Supplementary material for: Prevalence and Antibiotic Resistance Characteristics of Extraintestinal Pathogenic Escherichia coli among Healthy Chickens from Farms and Live Poultry Markets in China
Source: Animals (Basel). 2021 Apr 13;11(4):1112. doi: 10.3390/ani11041112 (PMC8070349; doi:10.3390/ani11041112)
Supplement: Supplementary file 1 [file animals-11-01112-s001.pdf]

Table S1 Origins of the samples collected in this study.

Location	Origin	Type of chickens	Farms/markets (no. of samples)	Year
Weifang in Shandong	White-feather broiler farm	White-feather broilers	Farm A (78)	2015
Hefei in Anhui	Layer farm	Brown-egg layers	Farm B (16)	2015
Liaocheng in Shandong	Layer farm	Brown-egg layers	Farm C (41)	2015
Binzhou in Shandong	Layer farms	Brown-egg layers	Farm D (86), Farm F (49)	2016
Xi'an in Shanxi	Layer farm	Brown-egg layers	Farm E (97)	2015
Qingdao in Shandong	Layer farms	Brown-egg layers	Farm G (37), Farm H (45)	2017
Linyi in Shandong	Live poultry market	Yellow-feather broilers	Market 1 (68)	2015
Qingdao in Shandong	Live poultry markets	Yellow-feather broilers	Market 2 (12), Market 3 (24), Market 4 (31), Market 7 (10), Market 8 (10), Market 9 (15), Market 10 (20), Market 11 (23), Market 12 (20), Market 13 (20), Market 14 (14)	2015
Yantai in Shandong	Live poultry markets	Yellow-feather broilers	Market 5 (23), Market 6 (32)	2015
Zaozhuang in Shandong	Live poultry market	Yellow-feather broilers	Market 15 (57)	2015
Zibo in Shandong	Live poultry market	Yellow-feather broilers	Market 16 (67)	2015
Weifang in Shandong	Live poultry market	Yellow-feather broilers	Market 17 (31)	2015
-	White-feather broiler farm		Farm A (78)	-
-	Layer farms		Farm B ~ Farm H (371)	-
-	Live poultry markets		Market 1~ Market 17 (477)	-
